# Engineered soluble, trimerized 4-1BBL variants as potent immunomodulatory agents

**DOI:** 10.1007/s00262-023-03474-8

**Published:** 2023-06-13

**Authors:** Claire Battin, Annika De Sousa Linhares, Judith Leitner, Anna Grossmann, Daniela Lupinek, Shiva Izadi, Alexandra Castilho, Petra Waidhofer-Söllner, Katharina Grabmeier-Pfistershammer, Jochen Stritzker, Peter Steinberger

**Affiliations:** 1 Themis Bioscience GmbH, Vienna, Austria; a subsidiary of Merck & Co., Inc., Rahway, NJ USA; 2 Loop Lab Bio GmbH, Vienna, Austria; 3 Division of Immune Receptors and T Cell Activation, Center for Pathophysiology, Infectiology, Institute of Immunology, Medical University of Vienna, Vienna, Austria; 4 Department of Applied Genetics and Cell Biology, Institute for Plant Biotechnology and Cell Biology, University of Natural Resources and Life Sciences, Vienna, Austria; 5 Center for Pathophysiology, Infectiology and Immunology, Institute of Immunology, Medical University of Vienna, Vienna, Austria

**Keywords:** Costimulation, Costimulation agonist, Immunotherapy, 4-1BB, TNFR-superfamily, T cell activation

## Abstract

Targeting co-stimulatory receptors promotes the activation and effector functions of anti-tumor lymphocytes. 4-1BB (CD137/TNFSF9), a member of the tumor necrosis factor receptor superfamily (TNFR-SF), is a potent co-stimulatory receptor that plays a prominent role in augmenting effector functions of CD8^+^ T cells, but also CD4^+^ T cells and NK cells. Agonistic antibodies against 4-1BB have entered clinical trials and shown signs of therapeutic efficacy. Here, we have used a T cell reporter system to evaluate various formats of 4-1BBL regarding their capacity to functionally engage its receptor. We found that a secreted 4-1BBL ectodomain harboring a trimerization domain derived from human collagen (s4-1BBL-Tri_XVIII_) is a strong inducer of 4-1BB co-stimulation. Similar to the 4-1BB agonistic antibody urelumab, s4-1BBL-Tri_XVIII_ is very potent in inducing CD8^+^ and CD4^+^ T cell proliferation. We provide first evidence that s4-1BBL-Tri_XVIII_ can be used as an effective immunomodulatory payload in therapeutic viral vectors. Oncolytic measles viruses encoding s4-1BBL-Tri_XVIII_ significantly reduced tumor burden in a CD34^+^ humanized mouse model, whereas measles viruses lacking s4-1BBL-Tri_XVIII_ were not effective. Natural soluble 4-1BB ligand harboring a trimerization domain might have utility in tumor therapy especially when delivered to tumor tissue as systemic administration might induce liver toxicity.

**Supplementary Information:**

The online version contains supplementary material available at 10.1007/s00262-023-03474-8.

## Introduction

T cells recognizing antigens depend on additional accessory signals to achieve full activation and to mount productive responses. Co-stimulation plays a major role in this process and consequently the enhancement of co-stimulatory signals is a promising strategy to strengthen T cell responses toward pathogens or tumor cells. Members of the tumor necrosis factor receptor superfamily (TNFR-SF) such as OX40, CD27, GITR and 4-1BB can co-stimulate the activation of T cells that recognize antigen. Among these receptors 4-1BB has the strongest capacity to enhance proliferation and cytokine production of human T cells [1]. 4-1BB is upregulated during T cell activation and engagement by its sole natural ligand 4-1BBL, a member of the tumor necrosis factor super family (TNF-SF), activates multiple signaling cascades involving TNF-receptor associated factors 1 and 2 (TRAF-1 and TRAF-2) [2–5]. TRAF-signaling activates the transcription factors NFκB and AP-1, which have central roles in T cell activation processes [2, 6]. 4-1BB signaling promotes T cell proliferation and cytokine production and induces anti-apoptotic molecules [1, 7–10]. Due to its exquisite capacity to augment effector functions and cytotoxic CD8^+^ T cell responses 4-1BB is considered a prime target for immunotherapy approaches to combat cancer cells [11–14]. Multiple reports have demonstrated that anti-4-1BB therapy can induce tumor regression in preclinical models [15–18]. 4-1BB signals were shown to overcome antigen-induced anergy and restore the function of exhausted CD8^+^ T cells [19, 20]. Several stimulatory 4-1BB antibodies have entered clinical trials with patients suffering from different types of cancer. However, the clinical development of 4-1BB antibodies has been slowed by severe side effects such a high-grade liver inflammation [14, 21, 22]. The interaction of antibody-based 4-1BB agonists with Fc $\gamma$-receptors (Fc $\gamma$ R) critically modulates their activity, which makes the in vivo function of antibody-based 4-1BB agonists hard to predict. Inter-individual differences in the expression of Fc $\gamma$ R classes and the existence of allelic Fc $\gamma$ R variants with altered antibody-binding capacity might contribute to this. Consequently, non-antibody-based 4-1BB agonists might be more viable alternatives to augment anti-tumor responses in cancer patients. Moreover, strategies aiming at introducing 4-1BB signals in the tumor microenvironment (TME) might enhance therapeutic effects while minimizing side-effects observed with the systemic use of 4-1BB agonists. This can be achieved by injecting 4-1BB agonits into the tumors or by employing bispecific constructs which co-target cancer-antigens and 4-1BB [23–27]. Using 4-1BB agonists as immunomodulatory payloads in anticancer vaccines or in tumor targeting virus vectors are especially promising approaches to exploit the potential of 4-1BB co-stimulation to enhance anti-tumor immunity while avoiding deleterious effects associated with the systemic introduction of 4-1BB signals.

Like most members of the TNF-SF, 4-1BBL is a type II transmembrane protein that is also present in soluble form. In contrast to soluble TNF, which can efficiently activate its receptor TNFR1, unmodified soluble 4-1BBL has minimal functional activity [28–30]. Thus, genetic engineering is required to render soluble 4-1BBL (s4-1BBL) functional. Here, we have generated and expressed various constructs encoding multimeric human 4-1BBL variants and assessed their capacity to function as receptor agonists in a highly sensitive T cell reporter system. Specifically, the introduction of a trimerization domain renders the s4-1BBL protein highly active in functionally engaging 4-1BB. In line with these data, we could confirm the co-stimulatory function of s4-1BBL-Tri_XVIII_ in primary T cell proliferation and activation assays. Importantly, cells infected with measles virus encoding s4-1BBL-Tri_XVIII_ efficiently produce agonistic 4-1BBL. Using a CD34^+^ humanized tumor mouse model, we could demonstrate that oncolytic measles viruses encoding s4-1BBL-Tri_XVIII_ significantly reduced tumor burden, whereas control measles viruses were not effective. Thus, we provide first evidence that constructs encoding the soluble 4-1BBL agonist can be used as immunomodulatory payloads in therapeutic viral vectors.

## Materials and methods

### Cell lines and antibodies

The African green monkey kidney cell line Vero 10–87 was obtained from WHO (world health organization, Geneva, Switzerland). The Jurkat cell line (JE6.1) and the mouse thymoma cell line BW5147 were derived from in house stocks and cultured in RPMI1640 supplemented with 10% FBS, penicillin (100 U/mL) and streptomycin (100 μg/mL) (from Sigma-Aldrich, St. Louis, MO). For transfection, HEK293T cells (kindly provided by A. Carmo, Porto, Portugal) were used. Mycoplasma contaminations were detected by using a recently described reporter system based on a human monocytic THP-1 cell line [31]. The following antibodies were purchased from Biolegend (San Diego, CA): 4-1BB (CD137)-PE (4B4-1), m4-1BB (CD137)-PE (17B5), 4-1BBL (CD137L)-PE (5F4), m4-1BBL (CD137L)-PE (TKS-1), CD14-APC (M5E2), CD4-BV421 (OKT4), CD8-PerCP (HIT8α), CD56-PE (5.1H11), CD56-BV510 (5.1H11), CD25-PeCy7 (M-A251). A DyLight-649-conjugated goat-anti-mouse IgG(H + L) antibody from Jackson ImmunoResearch (West Grove,PA) was used for anti-CD3 single chain fragment detection. The Strep-tag was detected by using the monoclonal mouse NWSHPQFEK-Tag (biotin) antibody (GeneScript, Leiden, The Netherlands) in conjunction with a Streptavidin-PE (Biolegend, San Diego, CA). The agonistic anti-human 4-1BB antibody urelumab (human, IgG4) was obtained from Creative Biolabs (Shirley, NY).

### Sample collection and PBMCs isolation

The study with peripheral blood mononuclear cells (PBMCs) was approved by the ethics committee of the Medical University of Vienna, Austria (ECS1936/2020). Heparinized whole blood (leucocyte reduction chambers) from healthy donors was purchased from the general hospital in Vienna (AKH; blood transfusion department). Using the standard density-gradient centrifugation using Lymphoprep solution (Technoclone, Austria), PBMCs were isolated and used for cellular assays.

### Mice

The in vivo experiment was carried out by TransCure bioServices SAS (Archamps, France) and approval was obtained from the local ethic committee (CELEAG) as well as the internal animal care and use committee of Merck & Co, Inc., Rahway, New Jersey, USA (Protocol Number:S-2024–301,194). Six-week-old female NOD-Prkdcem26Cd52Il2rgem26Cd22/NjuCrl mice were humanized by engraftment of 5–10 × 10^4^ hCD34^+^ hematopoietic stem cells isolated from human cord blood. Humanization rate was determined 12 weeks post-engraftment and only mice with > 25% of hCD45/mCD45 ratio entered the study. One week prior tumor cell engraftment, mice were enhanced for NK and myeloid cells by receiving a boost based on the transient expression of human cytokines IL-3, IL-4, IL-15 and GM-CSF. Thirty humanized mice were engrafted subcutaneously on the right flank with 3 × 10^6^ HCT-116 cells on day-15 and randomized into six treatment groups when an average tumor volume of 50–100 mm^3^ was reached (*n* = 5 mice per group): (1) Mock control (PBS), (2) Pembrolizumab, (3) TMV-018 (control measles virus), (4) TMV-018 + pembrolizumab, (5) TMV-110 (measles virus encoding s4-1BBL-Tri_XVIII_), (6) TMV-110 + pembrolizumab. The day of the first treatment was defined as experimental day 0 (D0). Measles viruses were administered weekly by intratumoral injection of 2 × 10^6^ TCID_50_/mouse for three weeks/three cycles. Pembrolizumab was administered weekly through intraperitoneal injection at a dose of 10 mg/kg. After the three cycles, pembrolizumab was continued weekly until the end of the study.

Mice were monitored for body weight and clinical signs 3 times per week. In addition, tumor volume was measured 3 times per week using a caliper. When large enough, the tumor volume was calculated using the formula: length × width^2^ × 0.52. Mice with a tumor volume exceeding 1000 mm^3^ or with a body weight loss > 20% were euthanized.

Data were analyzed with Graphpad Prism (version 9, GraphPad Software, Inc., La Jolla, CA). A Tukey test was performed after 2- way ANOVA to analyze differences in tumor volume, and differences in survival were analyzed with a Log-rank (Mantel cox) test.

### Construct design and cloning of soluble proteins

An expression plasmid encoding human 4-1BBL (Uniprot P41273) was used to generate the following 4-1BBL constructs: s4-1BBL, s-4-1BBL-Tri_XVIII_, s-4-1BBL-Tri_XVIII_^LL^, s-4-1BBL-Tri_XV_, s4-1BBL-TNC, triple-s4-1BBL, triple-s-4-1BBL-Tri_XVIII_^LL^ and s-4-1BBLsh-Tri_XVIII_. All constructs contain a CD5 Leader sequence followed by a Strep-Tag and a HIS-Tag sequence, separated by a GGCGG linker or 23-mer linker (LL or L*), upstream of the extracellular part of 4-1BBL (aa 85–254; Uniprot P41273). s4-1BBL-Tri_XVIII_, s4-1BBL-Tri_XV_, s-4-1BBL-Tri_XVIII_^LL^ and triple-s-4-1BBL-Tri_XVIII_^LL^ harbor a trimerization domain (human collagen XVIII; aa 1444–1501; Uniprot P39060.5 or XV NC1 domain; aa 1130–1188; Uniprot P39059) between the Tag sequences and the 4-1BBL extracellular region. Between the Tag sequences and the 4-1BBL extracellular region, the s4-1BBL-TNC construct has a trimerization domain chicken tenascin-C (TNC; aa 110–139; Uniprot P10039). The triple-s4-1BBL and triple-s-4-1BBL-Tri_XVIII_^LL^ constructs have three 4-1BBL (aa 85–254, different codons) separated by the addition of linker sequences (GGGGSGGGGSGGGGS).

### Cloning and rescue of oncolytic measles virus

The vector encoding for measles virus is based on a plasmid encoding the attenuated Schwarz strain of measles virus (Merieux, Sanofi-Pasteur Leimen, Germany) [32, 33]. An additional transcription unit (ATU) containing a CMV promoter was introduced between hemagglutinin (H) and large polymerase (L) protein at position number 3 (CMV3-ATU). The 4-1BBL-Tri_XVIII_ cassette harbors a kozak sequence, a CD5 leader sequence, a trimerization domain (human collagen XVIII) and the extracellular part of 4-1BBL (aa 85–254; Uniprot P41273) flanked by BsiWI/BssHII and was synthesized by GenScript, USA. For cloning, a CMV3-ATU backbone was linearized using restriction enzymes BsiWI/BssHII and ligated with 4-1BBL-Tri_XVIII_ template containing compatible ends. The resulting measles vector was named MV-4-1BBL-Tri_XVIII_ (TMV-110). As control, MV-SCD measles vector (TMV-018) encoding a super-cytosine-deaminase (SCD), a prodrug-converting enzyme was generated.

For rescue of oncolytic measles virus, confluent Vero cells (in 24 well plate) were transfected with 1 µg/well of vector DNA mixed with helper plasmids pcDIMER-N (100 ng/well), pcDIMER-P (20 ng/well), pcDIMER-L (100 ng/well) and FuGene HD (3 µl/µg DNA used). After 7 days, transfected Vero cells were harvested and overlaid on a fresh layer of Vero cells. After 7–10 days, syncytia were harvested for passage 0 (P0) production followed by clonal selection of single syncytia for viral stock preparation.

For cellular reporter assays, Vero cells were infected with TMV-018 and TMV-110 and supernatant was harvested. Oncolytic measles samples were filtered to remove virus and supernatant containing s4-1BBL-Tri_XVIII_ protein was used for further testing.

### Generation of cell lines

Jurkat NFκB-eGFP cell line and a triple parameter reporter cell line (NFκB-eCFP) were generated as previously described [34]. T cell stimulator cells (TCS) were engineered to express a membrane bound anti-human CD3 Ab single chain fragment (CD14 stem), which enables the triggering of the TCR-CD3 complex (“Signal 1”) [35]. 4-1BB and 4-1BBL were cloned into the retroviral expression vector pCJK2 and reporter cells and TCS were retrovirally transduced to express the respective molecules [36]. Further, cells were sorted to obtain high cell surface expression of the respective molecules with the SH800S Cell Sorter (Sony Biotechnology, San Jose, CA).

### Purification of proteins

Human s4-1BBL (aa 85–254; Uniprot P41273) fused to an N-terminal human CD5 leader for secretion and a 6xHIS-Tag upstream of the extracellular 4-1BBL region was cloned into the mammalian expression vector pCEP4. HEK293T cells were transiently transfected under reduced serum conditions (2% FCS). After 3 days, supernatants were harvested and dialyzed against 50 mM Na-Phosphate pH7.4, 150 mM NaCl prior to HisTALON purification. Column was equilibrated with 50 mM Na-Phosphate pH7.4, 150 mM NaCl and dialyzed cell culture supernatant was passed over the column twice, followed by washing with 50 mM Na-Phosphate pH7.4, 150 mM NaCl, 5 mM imidazole. Protein was then eluted in 50 mM Na-Phosphate pH7.4, 150 mM NaCl, 150 mM imidazole. Fractions of 0.5 ml were collected and measured. Protein containing fractions were pooled and dialyzed against Na-Phosphate pH7.4, 150 mM NaCl for imidazole removal. Finally, purified protein was analyzed by SDS-PAGE and Coomassie staining.

### Binding assays

For binding assays, 1 × 10^5^ 4-1BB expressing cells in a volume of 10 µl were incubated with 50 µl of 4-1BBL supernatants. As controls, cells that do not express 4-1BB, and a control-scFv, that also contains a Strep-Tag sequence, were used. After 30 min at 4 °C, the samples were washed in FACS-Buffer (1xPBS, 0.5% FCS, 0.005% NaN_3_) and stained with a biotinylated Strep-tag II mAb (GenScript, NJ) for 20 min on 4 °C. Afterward, samples were washed again and detection was performed by using Streptavidin-PE (BD Pharmingen, San Diego, CA). At last, samples were washed again and subsequently measured via flow cytometry, which was performed on a FACSCalibur flow cytometer (Becton Dickinson Immunocytometry System, San Jose, CA), using the CellQuest software. Data were analyzed with FlowJo (version 10.0.7, Tree Star, Ashland, OR) and Graphpad Prism (version 6, GraphPad Software, Inc., La Jolla, CA). Normalized binding was calculated by using the gMFI values of samples expressing 4-1BB and incubated with s4-1BBL protein and dividing with gMFI values of samples not incubated with s4-1BBL protein.

#### ELISA

ELISA plates were incubated overnight at 4 °C with a donkey anti-mouse IgG (3 μg/ml) (R&D Systems). Between each step, plates were washed 4 times with 200 μl of 1 × PBS. Subsequently, 1 μg/ml of Strep-Tag antibody (NWSHPQFEK unconjugated, 1 μg/ml; Genscript) was added and incubated for 2 h at RT. Plates were blocked with 1 × PBS, 3% BSA for 2 h at RT. The supernatants containing soluble 4-1BBL-Tri_XVIII_ and purified 4-1BBL-Tri_XVIII_ protein were coated at the indicated concentrations (diluted in 1 × PBS) for 2 h at 37 °C. Detection of coated proteins was performed with a biotinylated 4-1BBL antibody (polyclonal goat IgG; R&D Systems) in conjugation with a streptavidin-HRP (1:3000; Biolegend). Lastly, 3,3',5,5'-tetramethylbenzidine (TMB) was added and reaction was stopped after 5–10 min with 0.16 M sulfuric acid. OD 405 nm was determined using 650 nm as reference wavelength.

### Functional assays

To assess the functional activity of the 4-1BBL proteins, 4-1BB expressing reporter cells (5 × 10^4^) were co-cultured with control TCS (2 × 10^4^) in the presence or absence of supernatants containing soluble 4-1BBL or purified 4-1BBL proteins for 24 h at 37 °C with 5% CO_2_. TCS expressing 4-1BBL and the agonistic antibody urelumab (1 µg/ml) were used as positive control of 4-1BB co-stimulation. After 24 h, cells were harvested and stained with an anti-CD45.2-APC antibody to exclude TCS from Jurkat cells. Geometric mean of fluorescence intensity of viable reporter cells (mCD45.2‐APC negative) was used for further analysis. For some experiments, reporter gene activation in response to stimulation was normalized to control‐stimulated cells as indicated and expressed as fold induction.

### CFSE proliferation assays

1 × 10^7^ isolated PBMCs were labeled with CFSE (1 µl of 1 mM CFSE solution; C34554, Molecular Probes) for 4 min and processed as previously described [37]. Subsequently, 1 × 10^5^ CFSE-labeled PBMCs were co-cultured with UCHT1 or OKT3 (3 ng/ml or 300 ng/ml; Biolegend, San Diego, CA) in the presence or absence of different dilutions of supernatant containing s4-1BBL-Tri_XVIII_ protein. For all assays, 1 µg/ml urelumab was used as a positive control. After 5 or 7 days, PBMCs were harvested and proliferation (CFSE^low^ expression) and activation (CD25 expression) of CD4^+^ and CD8^+^ T cell subsets as well as of NK cells was assessed. Furthermore, supernatant of each co-cultures were harvested and cytokine profile was analyzed by Luminex multiplex cytokine analysis (System 100, Luminex Inc.). The concentration of IFN-*γ*, TNF-*α*, IL-13 and GM-CSF was measured according to the manufacturer’s instructions. CFSE^low^ and CD25 expression is depicted as fold induction (PBMCs stimulated with UCHT1/OKT3 in the presence of 4-1BBL protein is normalized to PBMCs stimulated with UCHT1/OKT3 alone). A single data point represents one donor of triplicates or duplicates.

## Results

### A trimerization domain confers potent agonistic properties to soluble 4-1BBL

**Fig. 1:**
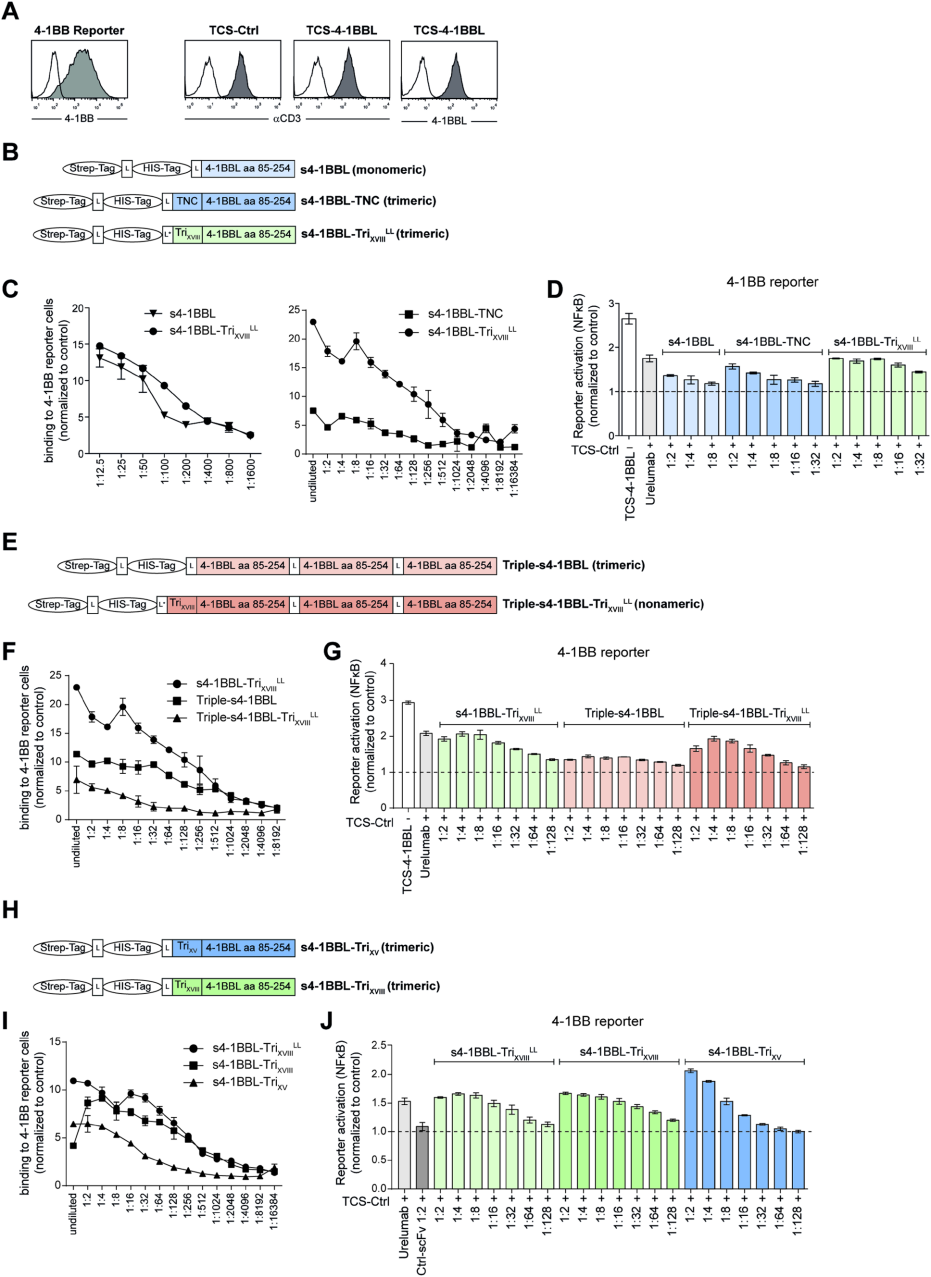
Multimerized s4-1BBL proteins exert potent co-stimulatory effects in 4-1BB expressing reporter cells. **a** Left: 4-1BB expression on control reporter cells (open histogram) and 4-1BB-reporter cells (grey histogram). Right: TCS control and TCS-4-1BBL were analyzed for expression of membrane-bound anti-CD3-scFv; 4-1BBL expression on control TCS (open histogram) and TCS-4-1BBL (grey histogram). **b**, **e**, **h** Schematic representation of synthetic genes encoding s4-1BBL, s4-1BBL-TNC, s4-1BBL-Tri_XVIII_^LL^ (long 23-mer linker; L*), Triple-s4-1BBL, Triple-s4-1BBL-Tri_XVIII_, s4-1BBL-Tri_XVIII_ (short GS linker L) and s4-1BBL-Tri_XV_. **c**, **f**, **i** 4-1BB reporter cells were probed with supernatants derived from HEK293T cells transfected with expression constructs encoding multimerized s4-1BBL proteins. Bound s4-1BBL was detected using a biotinylated Strep-tag II antibody in conjunction with streptavidin-PE via flow cytometry. Data are presented as fold induction relative to the values obtained upon binding of biotinylated Strep-tag II antibody on 4-1BB expressing reporter cells without soluble 4-1BBL protein (binding of s4-1BBL- Strep-tag II antibody/binding of Strep-tag II antibody (without s41BBL); MFI values are depicted). **d**, **g**, **j** 4-1BB reporter cells were stimulated with TCS control alone, in presence of urelumab (1 μg/ml), or cell culture supernatants containing s4-1BBL proteins at indicated dilutions. TCS expressing 4-1BBL served as additional positive control for 4-1BB activation. Activation of NFκB-eCFP was assessed by flow cytometry. Data are presented as fold change relative to the values obtained upon stimulation with TCS control alone (reporter cells stimulated with TCS-Ctrl in the presence of urelumab or s4-1BBLsh-Tri_XVIII_ supernatant/ reporter cells stimulated with TCS alone)

It is well described that for TNF family ligands, trimerization is essential for receptor triggering [23, 38]. Several studies have reported enhanced anti-tumor immunity and receptor activity upon oligomerizing proteins such as agonistic 4-1BB antibodies or generating trimers of CD27L, CD40L or 41BBL [23, 30]. Therefore, we introduced trimerization domains derived from human collagen (XV/XVIII NC1 domains) in the 4-1BBL molecule. We also generated a 4-1BBL variant containing a chicken tenascin-C (TNC) sequence which was previously shown to enhance the functional activity of various TNF ligand family members [30, 39]. To assess the capacity of the resultant soluble 4-1BBL proteins to bind and functionally engage the 4-1BB receptor we used a previously described Jurkat JE6-1-based triple parameter reporter (TPR) cell line expressing human 4-1BB [34] (Fig. 1a). Wildtype JE6-1-TPR (ctrl reporter), which do not express 4-1BB, served as a negative control. Within these experiments, our aim was to identify a 4-1BBL construct with high functional activity which is sufficiently strong expressed to yield cell culture supernatants which efficiently trigger 4-1BB signaling even in high dilutions. In a first step, we generated an expression construct (s4-1BBL) encoding peptide tags for detection and purification (Strep-tag and His-tag), and the ectodomain (amino acids 85–254) of human 4-1BBL separated by glycine-serine linkers. In addition to the monomeric version, we designed constructs encoding chicken tenascin-C (TNC) (s4-1BBL-TNC) [30] or a trimerization domain derived from human collagen (XVIII NC1 domain) with an extended 23-mer linker (s4-1BBL-Tri_XVIII_^LL^) upstream of the 4-1BBL sequence (Fig. 1b). HEK293T cells were transfected with constructs harboring s4-1BBL, s4-1BBL-TNC and s4-1BBL-Tri_XVIII_^LL^, and culture supernatants were harvested to assess the capacity of soluble 4-1BBL proteins to bind and functionally engage 4-1BB on the TPR cells. Cells were incubated with increasing concentrations of supernatant containing soluble 4-1BBL proteins and binding was detected via flow cytometry using a strep-tag antibody (Fig. 1c). We could detect strong interaction for both s4-1BBL and s4-1BBL-Tri_XVIII_^LL^ with cells expressing 4-1BB (Fig. 1c). Even at high dilutions of culture supernatants, binding signals could still be detected. In comparison to s4-1BBL-Tri_XVIII_^LL^, interaction of s4-1BBL-TNC protein to 4-1BB expressing cells was weaker, indicating a reduced binding efficiency or lower protein expression of s4-1BBL-TNC (Fig. 1c). For functional evaluation of the co-stimulatory capacity of s4-1BBL proteins, we co-cultured our reporter cells expressing 4-1BB with T cell stimulator cells (TCS), which display a membrane-bound anti-CD3-scFv and trigger the TCR/CD3 complex (“signal 1”) [35] (Fig. 1a). As a positive control for 4-1BB co-stimulation, we used TCS expressing the natural, membrane-bound ligand 4-1BBL (Fig. 1a). Upon TCR/CD3 stimulation, reporter cells are activated, and express eCFP or eGFP, which is under the control of NFκB responsive elements [34, 40]. 4-1BB signaling is a potent inducer of NFκB activation, and results in the upregulation of eCFP/eGFP expression in the reporter cells. Soluble 4-1BBL harboring the XVIII trimerization domain exerted potent co-stimulatory effects that were comparable to the agonistic 4-1BB antibody urelumab (1 µg/ml). In contrast, monomeric s4-1BBL failed to induce significant 4-1BB co-stimulation (Fig. 1d). Furthermore, supernatants containing s4-1BBL-TNC were less effective in co-stimulating 4-1BB reporter cells in comparison to s4-1BBL-Tri_XVIII_^LL^ (Fig. 1d).

Next, we generated a construct encoding three soluble 4-1BBL molecules connected via peptide linkers with and without an additional trimerization domain “Triple-s4-1BBL” and “Triple-s4-1BBL-Tri_XVIII_,” respectively (Fig. 1e). Supernatants from cultures expressing these constructs showed lower binding capacities to 4-1BB expressing cells than s4-1BBL-Tri_XVIII_^LL^ (Fig. 1f). This could potentially be owed to a poor expression of the triple construct, which was not detectable in Western blot (data not shown). Despite this, supernatants from cultures expressing the Triple-s4-1BBL-Tri_XVIII_^LL^ construct, which could potentially give rise to nonameric s4-1BBL, exerted potent co-stimulatory effects indicating a potent capacity of this s4-1BBL variant even at low concentrations (Fig. 1g).

Lastly, we replaced the long linker of s4-1BBL-Tri_XVIII_^LL^ upstream of the 4-1BB ectodomain by a short GS linker (s4-1BBL-Tri_XVIII_) and designed an additional construct where the XVIII NC1 trimerization sequence was replaced by a trimerization domain of type XV collagens (s4-1BBL-Tri_XV_) (Fig. 1h). Overall, all three proteins bound to 4-1BB expressing cells in a dose-dependent manner (Fig. 1i). The two new constructs s4-1BBL-Tri_XVIII_ and s4-1BBL-Tri_XV_ also potently co-stimulated the activation of NFκB in 4-1BB expressing reporter cells (Fig. 1j). Nevertheless, when tested at higher dilutions (1:64) their co-stimulatory capacity appeared to be lower than s4-1BBL-Tri_XVIII_^LL^. Taken together, our data clearly indicate that different formats of multimerized 4-1BBL can exert potent 4-1BB agonistic functions in a 4-1BB expressing reporter system. Since supernatants from cultures expressing s4-1BBL-Tri_XVIII_ with the short linker consistently demonstrated strong 4-1BB agonistic capacities, even at high dilutions, we focused on this format for further analysis. ELISA-based quantification indicated a strong expression of s4-1BBL-Tri_XVIII_ in HEK293T cells (Supplementary Fig. 1). To analyze the multimerization status of the proteins, s4-1BBL-Tri_XVIII_ and s4-1BBL were purified and analyzed by native gel electrophoresis and western blotting. In line with their capacity to trigger 4-1BB activation, the result of this experiment confirmed multimerization of s4-1BBL-Tri_XVIII_, but not of s4-1BBL, which was used as a control (Supplementary Fig. 2A, B). PGNase digestion experiments with s4-1BBL and s4-1BBL-Tri_XVIII_ indicated that both proteins were partially glycosylated (Supplementary Fig. 2C).

### Minimizing the ectodomain of 4-1BBL retains strong functional properties

**Fig. 2:**
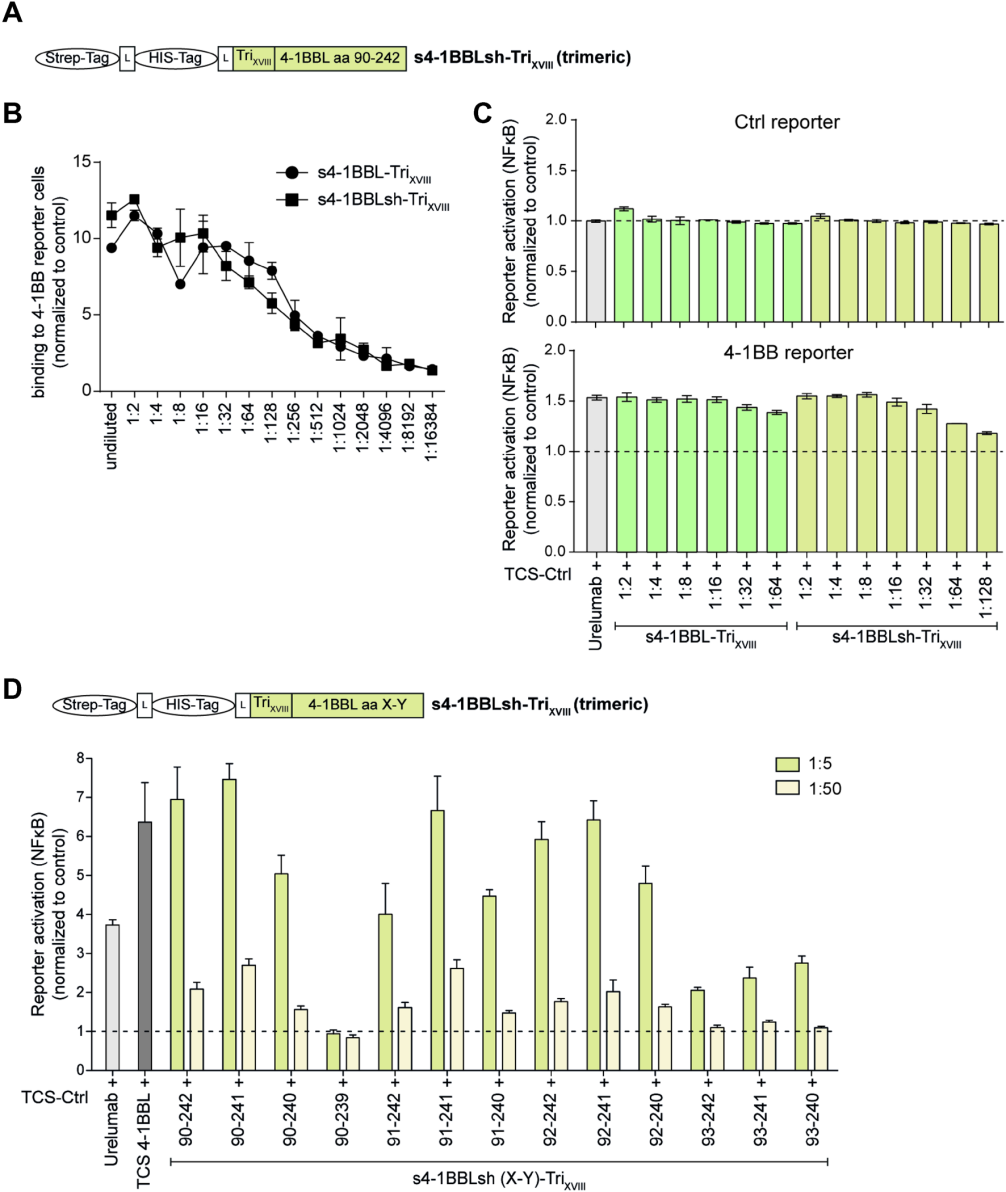
Shortened versions of 4-1BBL ectodomain retain strong agonistic effects **a** Schematic representation of a synthetic gene encoding a short version of trimerized s4-1BBL (s4-1BBLsh-Tri_XVIII_; aa 90–242). **b** 4-1BB reporter cells were incubated with supernatants containing s4-1BBL-Tri_XVIII_ and s4-1BBLsh-Tri_XVIII_ (90–242). Bound s4-1BBL was detected using a biotinylated Strep-tag II antibody in conjunction with streptavidin-PE. **c** 4-1BB reporter cells were stimulated with TCS control alone or in presence of urelumab (1 μg/ml) or cell culture supernatants containing s4-1BBL proteins used at indicated dilutions. TCS expressing 4-1BBL served as an additional positive control for 4-1BB activation. Data are presented as fold change relative to the values obtained upon stimulation with TCS control alone. **d** 4-1BB reporter cells were stimulated with TCS control alone or in presence of urelumab (1 μg/ml) or cell culture supernatants containing different short versions of s4-1BBLsh-Tri_XVIII_ (90–242, 90–241, 90–240, 90–239, 91–242, 91–241, 91–240, 92–242, 92–241, 92–240, 93–242, 93–241 and 93–240, respectively) proteins used at indicated dilutions. TCS expressing 4-1BBL served as an additional positive control for 4-1BB activation. Activation of NFκB-eGFP/ NFκB-eCFP was assessed via flow cytometry. Fold induction is depicted (reporter cells stimulated with TCS-Ctrl in the presence of urelumab or s4-1BBLsh-Tri_XVIII_ supernatant/reporter cells stimulated with TCS alone)

To identify the smallest polypeptide representing 4-1BBL with agonistic properties, we first generated a construct harboring amino acids 90–242 of 4-1BBL in the ectodomain sequence in comparison to 4-1BBL-Tri_XVIII_ containing amino acids 85–254 (Fig. 2a). Our experiments clearly indicate that a short 4-1BBLsh-Tri_XVIII_ (90–242aa) can also strongly interact in a dose-dependent manner to 4-1BB expressing cells (Fig. 2b). In addition, the 4-1BBLsh-Tri_XVIII_ (90–242aa) protein potently co-stimulated NFκB activation at a similar level as 4-1BBL-Tri_XVIII_ (85–254aa) in a functional 4-1BB reporter assay (Fig. 2c). To assess the shortest polypeptide still exerting strong co-stimulatory effects, we generated a series of truncated multimerized s4-1BBL variants (Fig. 2d). Our data demonstrated that a variant encoding aa 92–241 of the ectodomain of 4-1BB was still showing strong agonistic effects in 4-1BB expressing reporter cells. Variants 90/91/92-240 could still activate reporter cells but at somewhat reduced efficacy compared to their 90/91/92-241 counterparts. Further truncation on either end resulted in abrogation of stimulatory effects.

### s4-1BBL-Tri_XVIII_ potently co-stimulates the proliferation and activation of primary human CD4^+^ and CD8^+^ T cells

**Fig. 3:**
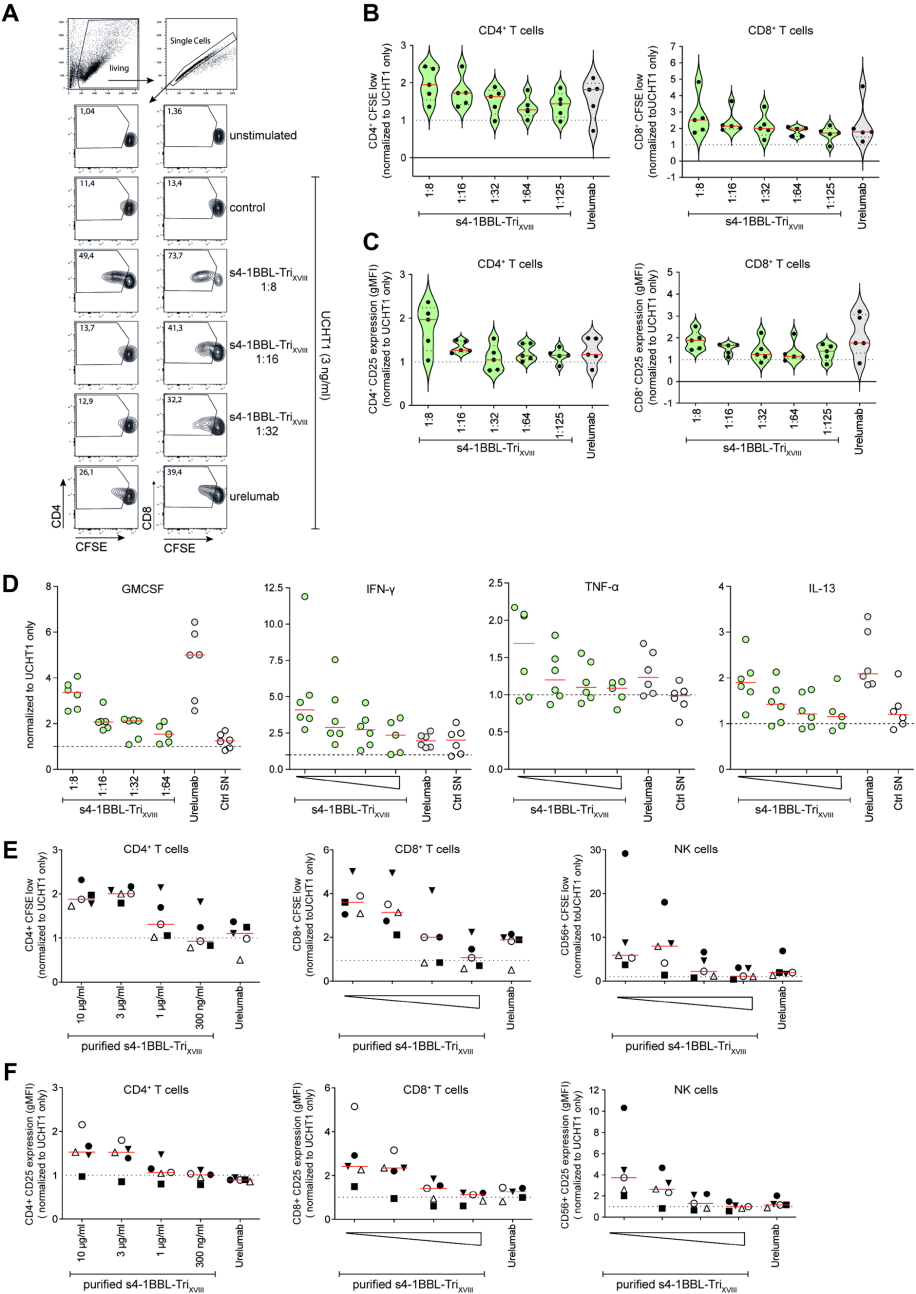
Soluble 4-1BBL-Tri_XVIII_ efficiently co-stimulates proliferation and activation of primary human CD4^+^ and CD8^+^ T cell (**a, b, c)** 1 × 10^5^ CFSE-labeled PBMCs were co-cultured with 3 ng/ml anti-human CD3 (UCHT1) in the absence or presence of different dilutions of culture supernatants containing s4-1BBL-Tri_XVIII_ or 1 µg/ml urelumab. After 5 days, PBMCs were harvested and stained for CD4, CD8, and CD25 and analyzed by flow cytometry. **a** Schematic representation of the gating strategy of one representative experiment is depicted. **b** T cell proliferation of 5 healthy volunteers is depicted. Data show percentages of CFSE^low^ gated on live CD4^+^ or CD8^+^ T cells normalized to 3 ng/ml of UCHT1 antibody alone. **c** T cell activation by analysis of geometric mean of fluorescence intensity (gMFI) of CD25 expression is shown for 5 healthy volunteers for the CD4^+^ and CD8^+^ T cell subsets. Data are normalized to values obtained to UCHT1 antibody alone. **d** Cell culture supernatants of PBMCs co-cultured with 300 ng/ml anti-human CD3 (OKT3) in the absence or presence of different dilutions of culture supernatants containing s4-1BBL-Tri_XVIII_ or 1 µg/ml urelumab were collected and analyzed by Luminex™-based multiplexing. Summary of the measured cytokines of 6 healthy volunteers are depicted for IFN-*γ*, GM-CSF, IL-13, and TNF-*α*. Data are normalized to OKT3 stimulation alone. Total of 6 donors is depicted. Supernatant of non-transfected HEK293 cells served as negative control. **e, f** 1 × 10^5^ CFSE-labeled PBMCs were cocultured with 3 ng/ml anti-human CD3 (UCHT1) in the absence or presence of different concentrations of purified s4-1BBL-Tri_XVIII_ and 1 µg/ml urelumab. T cell proliferation and T cell activation (CD25 expression) on CD4^+^ and CD8^+^ T cell subsets as well as on NK cells of 5 healthy donors is shown. Data are normalized to values obtained with UCHT1 alone. Each data point represents the mean of one donor measured in triplicates/duplicates. Quantification of s4-1BBL in cell culture supernatants is shown in supplementary Fig. 1

4-1BB has a prominent co-stimulatory function on the CD8^+^ T cell subsets, but also on CD4^+^ T cells, B cells and NK cells [12, 14]. We isolated PBMCs from healthy donors and analyzed the co-stimulatory capacity of s4-1BBL-Tri_XVIII_ protein to activate and induce proliferation in primary human T cell subsets. CFSE-labeled PBMCs were co-cultured with anti-CD3 antibodies (3 ng/ml UCHT1) in the presence or absence of culture supernatants containing s4-1BBL-Tri_XVIII_ protein (in different dilutions) (Fig. 3a). Urelumab (1 µg/ml) was used as positive control. After 5 days of co-culture, proliferation and the expression of the activation marker CD25 was analyzed on CD4^+^ and CD8^+^ T cell subsets. We observed a dose-dependent co-stimulation of s4-1BBL-Tri_XVIII_ on proliferation and CD25 upregulation in the CD4^+^ and CD8^+^ T cell subsets, which was comparable to urelumab (1 µg/ml) (Fig. 3a–c). The strongest co-stimulatory effect on proliferation and CD25 upregulation was detected upon addition of s4-1BBL-Tri_XVIII_ supernatants at a final dilution of 1:8. Moreover, supernatants of co-cultures of PBMCs stimulated with anti-CD3 antibodies (300 ng/ml OKT3) in the presence or absence of culture supernatants containing s4-1BBL-Tri_XVIII_ or urelumab were harvested at day 5 and the cytokine pattern was assessed using Luminex™-based multiplexing. Cytokine analysis revealed that s4-1BBL-Tri_XVIII_ potently and dose-dependently co-stimulated the production of cytokines such as GM-CSF, TNF- *α*, IFN- *γ* and IL-13 (Fig. 3d). Similar levels were obtained for the agonistic 4-1BB antibody urelumab, even though some differences were observed, particularly for IFN-*γ* (higher in presence of s4-1BBL-Tri_XVIII_) and GM-CSF (higher in presence of urelumab) production. Lastly, we determined the co-stimulatory function of purified s4-1BBL-Tri_XVIII_ protein on primary human CD4^+^ and CD8^+^ T cell subsets as well as on NK cells. Again, CFSE-labeled PBMCS were incubated with anti-CD3 antibodies (3 ng/ml UCHT1) in the absence or presence of increasing concentrations (300 ng/ml to 10 µg/ml) of the purified protein for 7 days. Purified s4-1BBL-Tri_XVIII_ strongly induced proliferation and upregulated CD25 expression in the CD4^+^ and CD8^+^ T cells as well as in NK cells in a dose-dependent manner (Fig. 3e, f).

### Assessment of soluble murine 4-1BBL (ms4-1BBL-Tri_XVIII_*)*

**Fig. 4:**
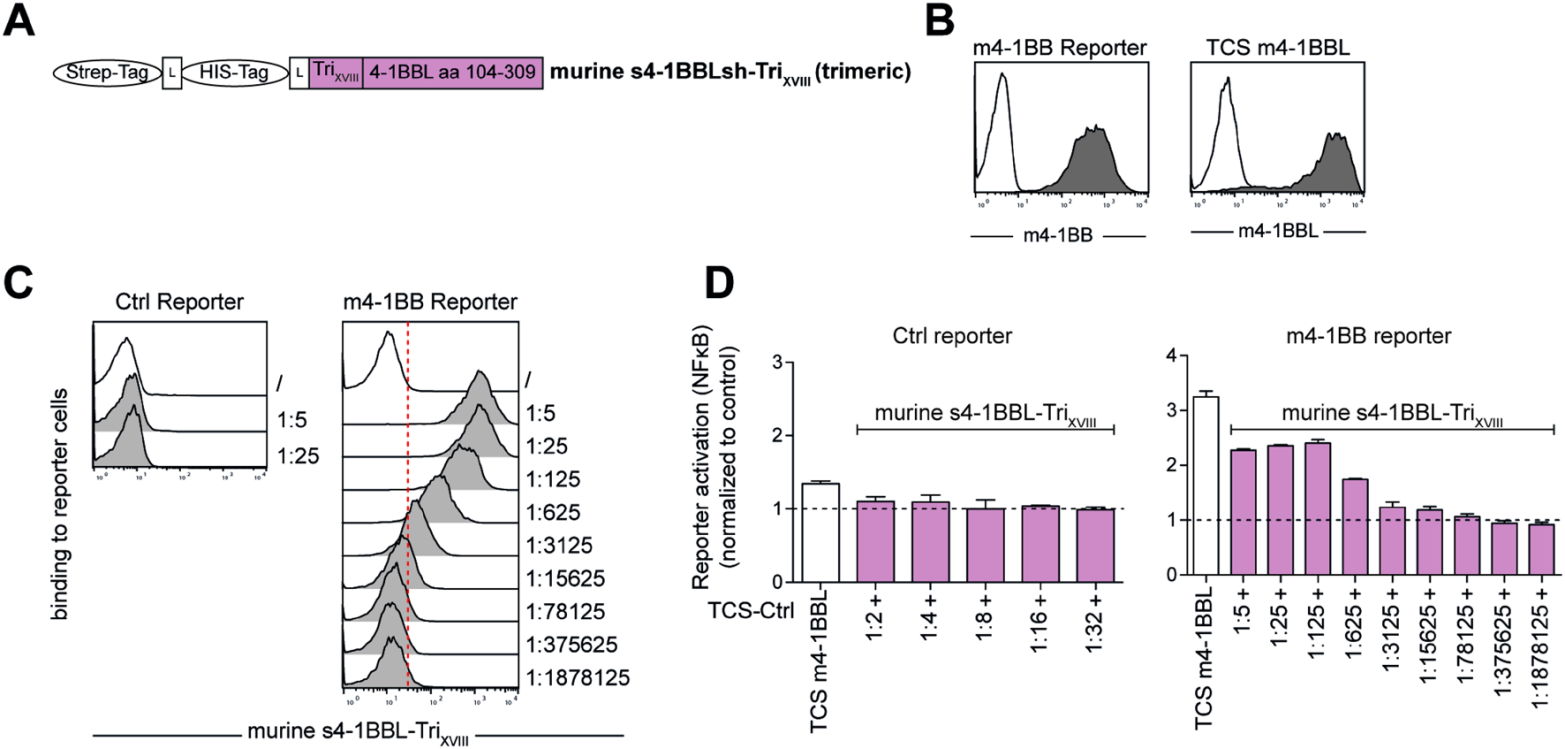
A murine 4-1BBL-Tri_XVIII_ protein strongly co-stimulates reporter cells expressing m4-1BB **a** Schematic representation of a synthetic gene encoding murine s4-1BBL-Tri_XVIII_ (ms4-1BBL-Tri_XVIII_) that was cloned into the mammalian expression vector pCEP4. **b** Analysis of cell surface expression of murine 4-1BB on reporter cells and murine 4-1BBL on T cell stimulator cells (TCS). **c** Control (Ctrl) reporter cells and m4-1BB reporter cells were probed with diluted supernatants derived from HEK293T cells transfected with expression constructs encoding ms4-1BBL-Tri_XVIII_. Bound ms4-1BBL-Tri_XVIII_ was detected using a biotinylated Strep-tag II antibody in conjunction with streptavidin-PE. **d**, **e** Control reporter cells and m4-1BB reporter cells were stimulated with TCS alone or with TCS in the presence of cell culture supernatants containing ms4-1BBL-Tri_XVIII_ used at indicated dilutions. TCS expressing m4-1BBL served as positive control for m4-1BB activation. Data of one representative experiment is shown

To characterize the co-stimulatory function of multimerized murine 4-1BBL, an expression construct encoding soluble murine s4-1BBL-Tri_XVIII_ (ms4-1BBL-Tri_XVIII_) was generated. Again, this construct harbors a CD5 leader sequence followed by a Strep-Tag and a HIS-Tag sequence, separated by a GGCGG linker, upstream of the extracellular part of murine 4-1BBL (aa 104–309; UniProt P20334; (Fig. 4a). Like the human s4-1BBL-Tri_XVIII_ construct, murine s4-1BBL-Tri_XVIII_ also contains a trimerization domain between the Tag sequences and the m4-1BBL ectodomain (Fig. 4a). For the evaluation of ms4-1BBL-Tri_XVIII_ protein, we generated reporter cells expressing murine 4-1BB and TCS expressing murine 4-1BBL to be used as a positive control (Fig. 4b). Ms4-1BBL-Tri_XVIII_ was expressed in HEK293 cells and reporter cells expressing mouse 4-1BB were probed with culture supernatants containing soluble ms4-1BBL-Tri_XVIII_ proteins. Results indicated a strong and specific interaction of ms4-1BBL-Tri_XVIII_ with cells expressing mouse 4-1BB and binding signals were detected at dilutions up to 1:15,625 (Fig. 4c). Ms4-1BBL-Tri_XVIII_ also very potently co-stimulated the activation of mouse 4-1BB reporter cells (Fig. 4d). Taken together our data indicate that ms4-1BBL-Tri_XVIII_, in analogy to our human construct, is strongly expressed and functions as a potent agonist for m4-1BB co-stimulation.

### Supernatants derived from cells infected with oncolytic measles virus vectors encoding soluble s4-1BBL-Tri_XVIII_ induce potent 4-1BB co-stimulation

**Fig. 5:**
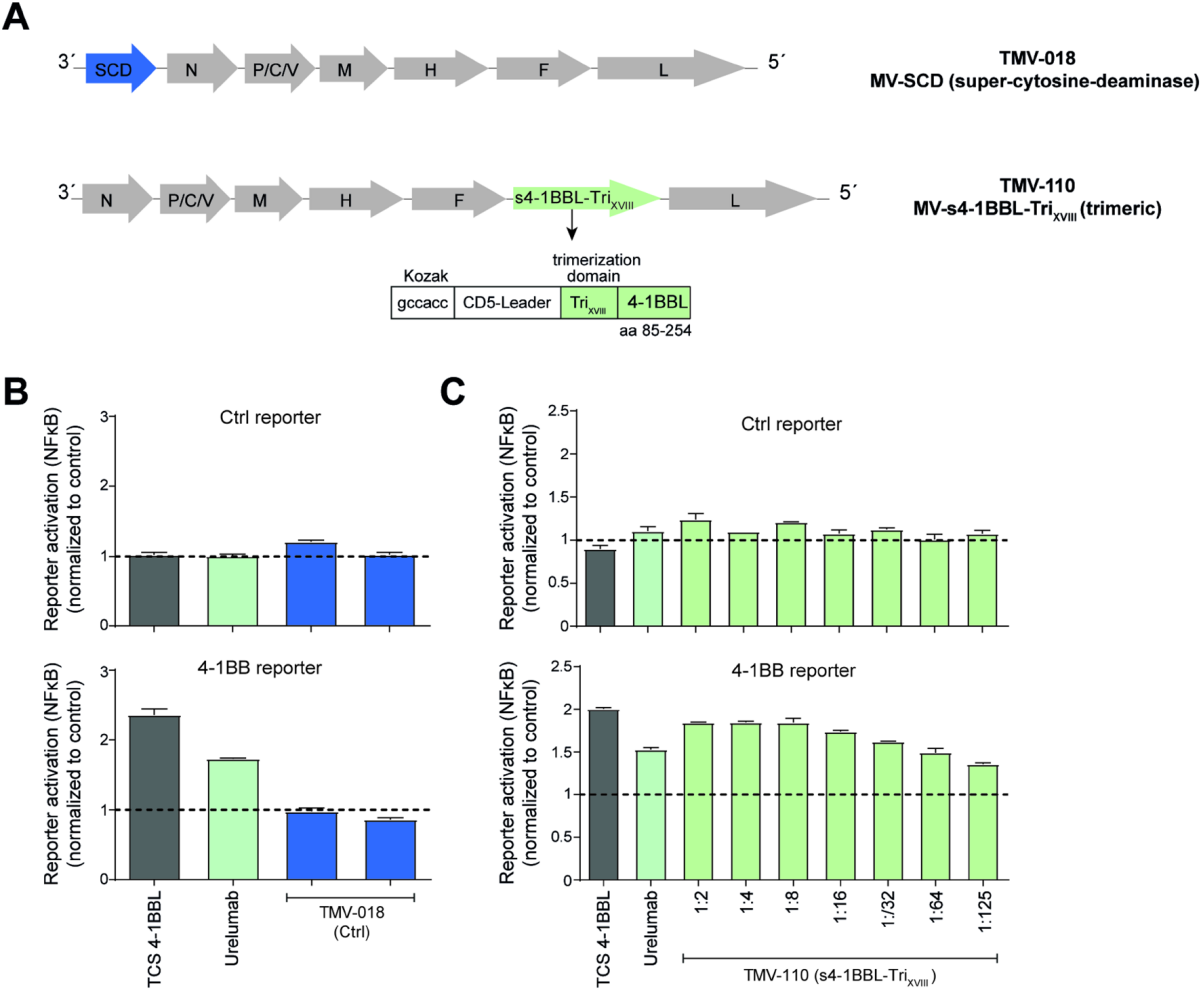
Assessment of co-stimulatory capacity of supernatants derived from cells infected with measles vectors encoding soluble 4-1BBL **a** Schematic representation of oncolytic measles viral vector encoding human s4-1BBL-Tri_XVIII_. **b**, **c** Control (Ctrl) reporter cells and 4-1BB reporter cells were probed with diluted supernatants derived from cells infected with control measles virus (TMV-018), or virus encoding human s4-1BBL-Tri_XVIII_ (TMV-110). TCS expressing 4-1BBL as well as urelumab (1 μg/ml) in combination with TCS ctrl served as positive control for 4-1BB activation. Activation of NFκB-eCFP was assessed via flow cytometry. Data of one representative experiment is shown. Data are presented as fold induction relative to the values obtained upon stimulation with TCS control alone (reporter cells stimulated with TCS-Ctrl in the presence of Urelumab or TMV-110 (s4-1BBLsh-TriXVIII) supernatant or TMV-018 supernatant divided by reporter cells stimulated with TCS alone (control)). N, nucleocapsid gene; P, phosphoprotein gene; C/V, non-structural proteins, M, matrix protein gene; F, fusion protein gene; H, hemagglutinin gene; L, large protein gene

Soluble 4-1BBL agonists could potentially be used as synergistic immunomodulatory payloads in therapeutic viral vectors. To test the feasibility of such an approach, a gene encoding s4-1BBL-Tri_XVIII_ was incorporated into an oncolytic measles virus vector (TMV-110) (Fig. 5a). Supernatants derived from Vero cells infected with control measles virus vectors (TMV-018, MV-SCD) and virus vector encoding s4-1BBL-Tri_XVIII_ (TMV-110, MV- s4-1BBL-Tri_XVIII_) were collected, and functional assays were performed using human 4-1BB expressing reporter cells in combination with TCS-Ctrl. We could demonstrate strong dose-dependent 4-1BB agonistic activities and a potent co-stimulation of 4-1BB expressing T cell reporter cells for supernatants of TMV-110 infected cells, which was not the case for control supernatant of TMV-018 infected cells (Fig. 5b, c). These data clearly indicate that measles virus vectors can be engineered to deliver payloads that have the capacity to enhance T cell and NK cell responses via 4-1BB co-stimulation.

### Oncolytic measles viruses encoding soluble s4-1BBL-Tri_XVIII_ delay tumor growth in a CD34^+^ humanized mouse model

**Fig. 6:**
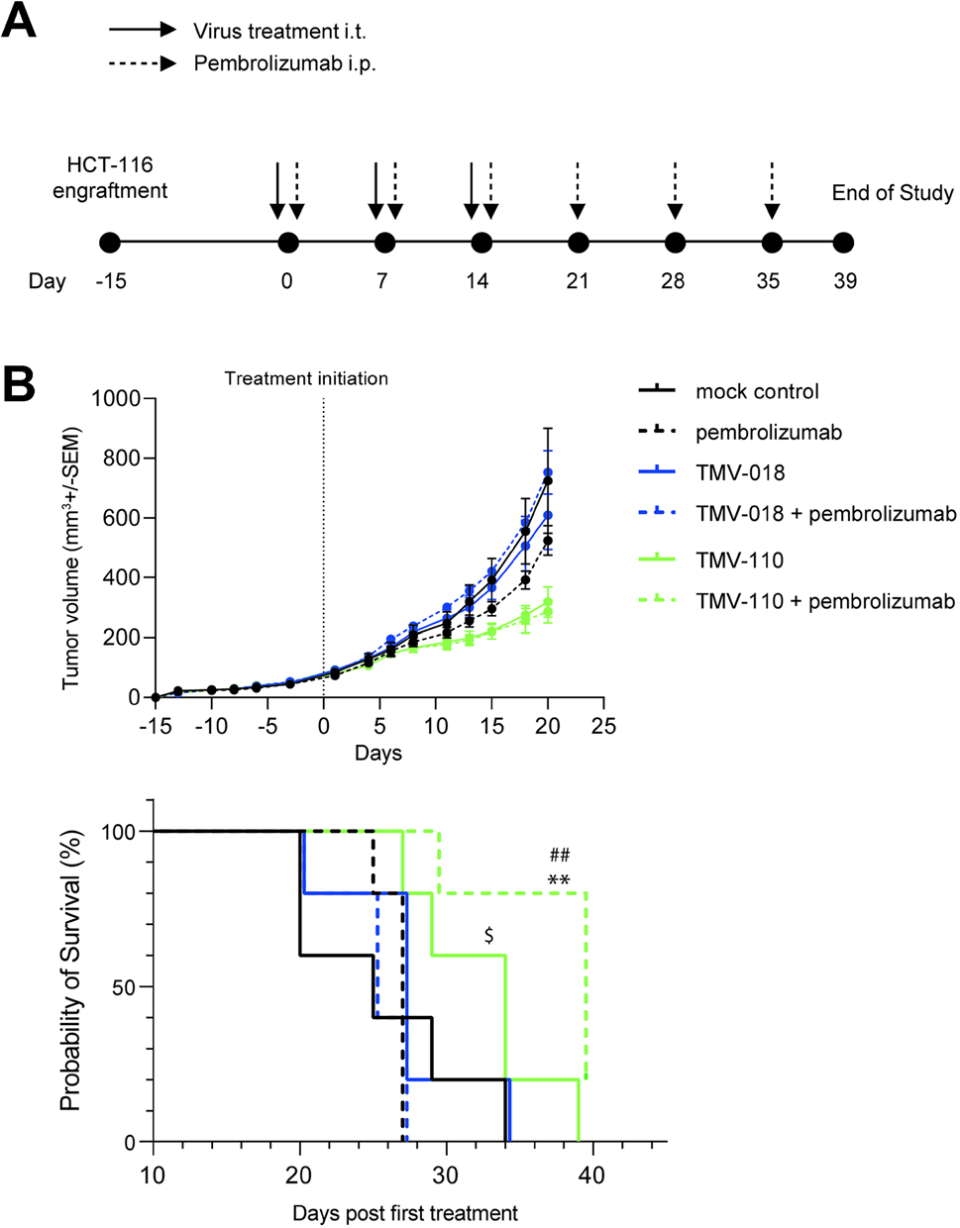
Efficacy of an oncolytic measles virus expressing s4-1BBL-Tri_XVIII_ in a CD34 + humanized mouse tumor model **a** Top: Experimental study design depicting the treatment of TMV-018 (MV-SCD; Control)/TMV-110 (MV-s4-1BBL-Tri_XVIII_) as monotherapy and in combination with pembrolizumab in a CD34^+^ humanized mouse tumor model. **b** Average tumor volume form D0 to D20 is shown (tumor volume expressed in mm.^3^). *N* = 5 mice per group. Means ± SEM is shown. A Two-way ANOVA followed by a Tukey post-test was used to compare the treatment groups. Mock control versus TMV-110 (***), mock control versus TMV-110 + pembrolizumab (***), pembrolizumab versus TMV-018 + pembrolizumab (***), pembrolizumab versus TMV-110 + pembrolizumab (*), # TMV-018 versus TMV-110 (***), TMV-018 versus TMV-110 + pembrolizumab (***), TMV-018 + pembrolizumab versus TMV-110 (***), TMV-018 + pembrolizumab versus TMV-110 + pembrolizumab (***). ns: *p* > 0.12, * *p* < 0.033, ** *p* < 0.002, *** *p* < 0.001. Bottom: Percentage of survival of TMV-110 alone or in combination with pembrolizumab. Log-rank Mantel Cox test was used to compare survival curves. *TMV-110 + pembrolizumab versus mock control. #TMV-110 + pembrolizumab versus pembrolizumab. $ TMV-110 versus pembrolizumab. $ *p* < 0.05; ##, ** *p* < 0.01

A CD34^+^ humanized mouse tumor model was used to evaluate whether TMV-110 can augment anti-tumor immunity in vivo. Prior treatment, mice were engrafted with HCT-116 tumor cells at D-15. After tumor volume reached 50–100 mm^3^, mice were treated with intratumoral mock, control oncolytic measles viruses (TMV-018, 2 × 10^6^ TCID_50_/mouse), or oncolytic measles viruses encoding 4-1BBL-Tri_XVIII_ (TMV-110, 2 × 10^6^ TCID_50_/mouse). Virus was administered weekly, either alone or in combination with the PD-1 blocking antibody pembrolizumab for a total of 3 cycles, and pembrolizumab was continued weekly until the end of the study (Fig. 6a). Treatment with TMV-018 had only marginal effects, whereas treatment with TMV-110 led to a significant reduction in tumor growth. Compared to pembrolizumab treatment, the reduction observed upon administration of TMV-110 was more pronounced (pembrolizumab vs. TMV-110 + pembrolizumab, *p* ≤ 0.033) (Fig. 6b top). Treatment with TMV-110 alone or in combination with pembrolizumab significantly prolonged the survival of the tumor bearing animals (median survival for control or pembrolizumab was 25 and 27 days, respectively, vs. 34 days for TMV-110 and 39 days for TMV-110 + pembrolizumab) (Fig. 6b bottom). All in all, combination with oncolytic measles viruses and immune checkpoint inhibitors may represent novel treatment in remodeling the anti-tumor response.

## Discussion

4-1BB signaling potently augments the activity of cytotoxic effector cells that have central roles in tumor immunity which make this receptor a highly promising therapeutic target. However, the use of the agonistic 4-1BB antibodies in clinical trials was associated with severe side effects. Fc-receptor-mediated enhancement of 4-1BB agonism has been proposed to critically contribute to on target off tumor toxicities of 4-1BB antibodies [24, 41]. 4-1BB agonists that trigger co-stimulation independent of Fc-receptors are promising alternatives to classical antibodies. Various formats have been developed and were shown to have efficacy in vitro and in preclinical models. Among these are bispecific antibodies co-targeting 4-1BB with PD-L1 or B7-H3, which are both immunomodulatory molecules expressed in tumors [26, 27]. Trimerized bispecific antibodies co-targeting 4-1BB and human EGFR induced tumor regression in mice inoculated with hEGFR^+^ tumor cells and this treatment was not associated with significant toxicity [23]. The 4-1BB/HER2 bispecific antibody-anticalin fusion PRS-343 was demonstrated to induce growth-inhibition of HER2 positive tumor cells in vivo and infusion of this bispecific agent into cynomolgus monkeys was well tolerated. Phase I clinical trials with PRS-343 alone or in combination with a PD-1 blocker have been initiated (NCT03330561 and NCT03650348) [25]. Claus et al. have generated and successfully validated tumor targeted 4-1BB agonists harboring 4-1BBL and Fabs binding to fibroblast-activating protein (FAB) expressed on tumor stroma cells or to CD19 [24].

Here, we describe the development of 4-1BB agonists that are based on soluble 4-1BBL and function independent of tumor antigens or Fc-receptors. We demonstrate that s4-1BBL harboring trimerization domains, but also three soluble 4-1BBL molecules linked via a peptide linker can potently trigger the activation of T cell reporters expressing 4-1BB. A potential limitation of our study is that we have not characterized the different 4-1BBL proteins in detail and did not compare their functional activity at the same concentrations. Instead, we compared supernatants of HEK cells expressing various constructs generated under same conditions with the aim to identify a protein which has the potential to be used as a immunostimulatory payload in viral vector vaccination. Our experiments indicate a high potential of 4-1BBL-fused to a collagen XVIII trimerization domain (s4-1BBL-Tri_XVIII_). Compared to 4-1BBL fused to a trimerization motif derived from tenascin C (TNC), s4-1BBL-Tri_XVIII_ appeared to have a stronger capability to functionally engage 4-1BB. Moreover, in contrast to the chicken-derived tenascin C sequence, s4-1BBL-Tri_XVIII_ contains only human sequences and is therefore less likely to induce anti-drug antibodies. In addition, we have generated and tested several truncated 4-1BBL molecules and found that s4-1BB-Tri_XVIII_ containing amino acids 92–241 of 4-1BBL represents a shortened version with strong agonistic activity. Furthermore, s4-1BBL-Tri_XVIII_ and the 4-1BB agonistic antibody urelumab were similarly potent in co-stimulating the activation of human CD4 and CD8 T cells.

Studies in cancer models have demonstrated that 4-1BB agonists can promote anti-tumor immunity [15–17, 42]. However, 4-1BB triggering can cause undesired side-effects. In mice, 4-1BB agonistic antibodies were demonstrated to cause polyclonal activation of CD8^+^ T cells, production of proinflammatory cytokines as well as immune-related abnormalities affecting spleen, bone marrow and liver [43–45]. The treatment with 4-1BB agonists led to mononuclear inflammation driven by antigen-independent activation of non-tumor-specific CD8^+^ T cells [44, 45]. Moreover, recent work in our laboratory indicates that in the context of antigen-specific CD8^+^ T cell responses the presence of 4-1BB co-stimulation promotes the activation of bystander CD8^+^ T cells and NK cells [46]. Thus, there is ample evidence beyond the adverse outcomes of clinical trials with urelumab that systemic administration of 4-1BB agonists should be done with utmost care.

Here, we propose that oncolytic measles vectors encoding s4-1BBL-Tri_XVIII_ (MV-s4-1BBL-Tri_XVIII_) have utility to function as immunomodulatory payloads specifically by infecting and killing malignant cells as well as activating T cells. By employing the reverse genetic system, genetically engineered oncolytic measles virus can be rescued from cloned cDNA [47]. For this study, oncolytic measles vectors encoding the transgene s4-1BBL-Tri_XVIII_ were generated and rescued on Vero cells. Culture supernatants derived from cells infected with MV-s4-1BBL-Tri_XVIII_ exerted potent 4-1BB agonistic properties in a T-cell-based reporter system. Furthermore, oncolytic MV-s4-1BBL-Tri_XVIII_ was evaluated in vivo using a CD34^+^ humanized mouse tumor model. Importantly, compared to control measles virus, MV-s4-1BBL-Tri_XVIII_ (as well as combination therapy with MV-s4-1BBL-Tri_XVIII_ + pembrolizumab) was efficient in reducing tumor growth and prolonging the survival of tumor bearing mice. Introducing sequences encoding non-engineered, membrane-bound 4-1BBL might also enhance the efficacy of oncolytic MV, but in contrast to soluble trimeric 4-1BBL the immune stimulatory effect of membrane-bound 4-1BBL will be confined to cells that have direct contact with the MV-infected cells. Several studies as well as clinical trials have already shown the oncolytic potential of engineered measles virus retargeting the virus to tumor-specific ligands/antigens like CD20, CD38 or carcinoembryonic antigen (CEA) [48, 49]. Veinalde et al. have generated MV vectors encoding monoclonal antibodies (nivolumab, pembrolizumab and atezolizumab) targeting the human PD-1/PD-L1 receptors and could observe an increase in T-cell effector cytokines as well as tumor-specific T cell memory [47, 50, 51]. In summary these data indicate that immunomodulatory payloads have the potential to increase that efficacy of oncolytic virus treatments. Its potent co-stimulatory activities make s4-1BBL-Tri_XVIII_ an especially promising candidate for such approaches.

### Supplementary Information

Below is the link to the electronic supplementary material.

Supplementary file1 (PDF 195 kb)

## Data Availability

The datasets generated during and/or analyzed during the current study are available from the corresponding author on reasonable request.
