# Supplementary material for: Engineered soluble, trimerized 4-1BBL variants as potent immunomodulatory agents
Source: Cancer Immunol Immunother. 2023 Jun 13;72(9):3029–43. doi: 10.1007/s00262-023-03474-8 (PMC10412504; doi:10.1007/s00262-023-03474-8)
Supplement: Supplementary file 1 — Supplementary file1 (PDF 195 kb) [file 262_2023_3474_MOESM1_ESM.pdf]

# Supplementary Figures

## Quantification of s4-1BBL-Tri$_{XVIII}$ in cell culture supernatants

To quantify the amount of s4-1BBL-Tri$_{XVIII}$ in culture supernatants of transfected HEK293T cells, we performed binding assays based on ELISA. The cell culture supernatants and the purified protein were added to strep-tag coated plates and binding was detected with a biotinylated 4-1BBL antibody in conjugation with streptavidin-HRP. We observe a strong dose dependent binding for both proteins. Based on these data, we can conclude that culture supernatants of HEK293T cells transfected with the s4-1BBL-Tri$_{XVIII}$ construct contain high amounts of s4-1BBL-Tri $_{XVIII}$.

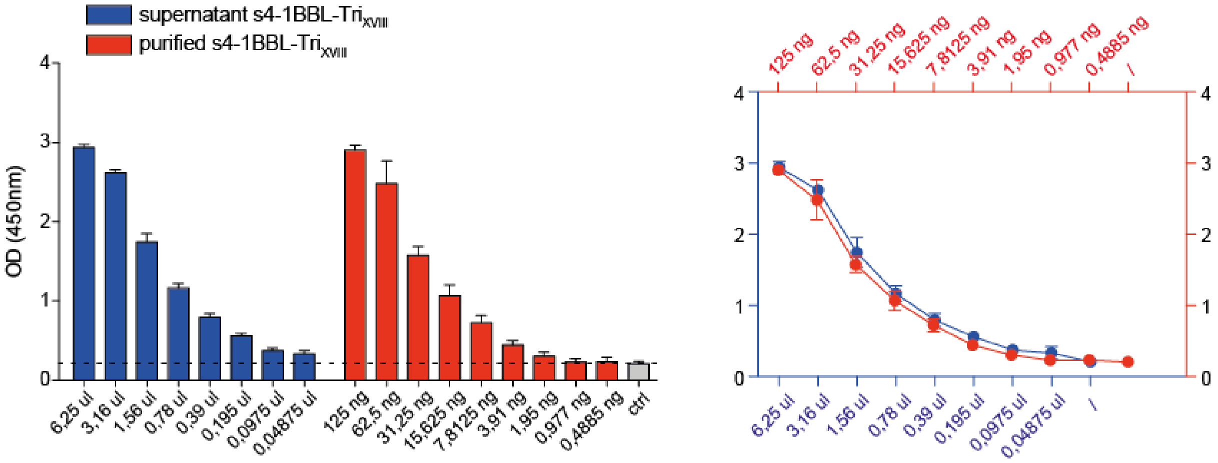


**Supplementary Figure 1 Quantification of s4-1BBL-TriXVIII in cell culture supernatants**

Binding of s4-1BBL-Tri$_{XVIII}$ protein (supernatant and purified) was analyzed by ELISA at different diluations and concentrations, respectively. Two experiments performed in duplicate are depicted.

## Multimerization status of s4-1BBL-Tri$_{XVIII}$ and s4-1BBL

Highly sensitive JE6-1-NF-kB::eGFP-4-1BB reporter cells were used to test supernatants derived from HEK cells expressing s4-1BBL-Tri$_{XVIII}$ and s4-1BBL regarding their costimulatory activity (Supplementary Fig. 2A). To analyze their multimerization status s4-1BBL-Tri$_{XVIII}$ and s4-1BBL proteins purified via Streptactin affinity chromatography were analyzed by native gel electrophoresis and western blotting. An Streptag antibody was used for detection of proteins (Supplementary Fig. 2B). To analyze the glycosylation status of the s4-1BBL-Tri$_{XVIII}$ and s4-1BBL proteins they were treated with PGNase. Together with nontreated samples, they were

separated by SDS-PAGE and subjected to western blotting. A streptag antibody was used for detection (Supplementary Fig. 2C).

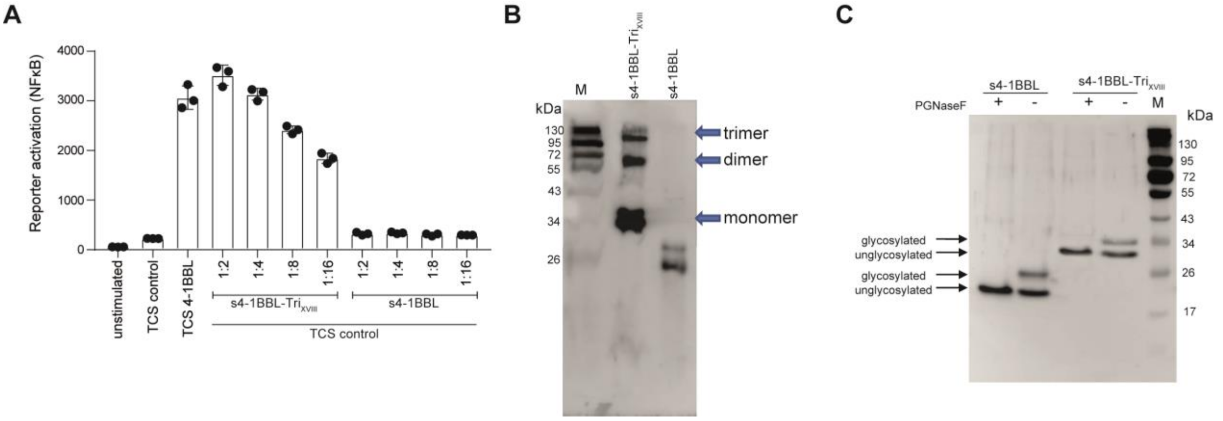


**Supplementary Figure 2 Multimerization and glycosylation of s4-1BBL-TriXVIII and s4-1BBL proteins.**

A) Supernatants of HEK293T cells expressing the indicated constructs were tested for their costimulatory activity using 4-1BB reporter cells stimulated with TCS-control. TCS expressing 4-1BBL served as positive control. B) Purified s4-1BBL-Tri$_{XVIII}$ and s4-1BBL proteins separated by native gel electrophoresis were analyzed by western blotting. A Streptag antibody was used for detection. C) PGNase F-treated and nontreated s4-1BBL-Tri$_{XVIII}$ and s4-1BBL proteins were separated by SDS-PAGE and subjected to western blotting. A streptag antibody was used for detection.
